# Acceptance of Unsupervised App-Based Cognitive Assessment in Outpatient Care: An Implementation Study

**DOI:** 10.2196/62706

**Published:** 2025-02-13

**Authors:** Iris Blotenberg, Melanie Boekholt, Nils Lieberknecht, Paula Säring, Jochen René Thyrian

**Affiliations:** 1 German Center for Neurodegenerative Diseases (DZNE), Interventional Health Care Research, Ellernholzstr. 1-2, Greifswald, 17489, Germany, 49 3834-86-19534; 2 neotiv GmbH, Magdeburg, Germany; 3 Institute for Community Medicine, University Medicine Greifswald, Greifswald, Germany

**Keywords:** mild cognitive impairment, Alzheimer disease, dementia, cognition, computerized assessment, digital assessment, digital cognitive biomarkers, home-based assessment, digital platform, mobile phone

## Abstract

**Background:**

The use of unsupervised digital cognitive assessments provides considerable opportunities for early and comprehensive testing for Alzheimer disease, minimizing the demand on time and personnel resources in medical practices. However, the acceptance within health care has yet to be assessed.

**Objective:**

In this implementation study, the acceptance of an app-based, repeated cognitive assessment for early symptoms of Alzheimer disease in the outpatient care setting from both physicians’ and patients’ perspectives was examined.

**Methods:**

In total, 15 primary care practices participated, where patients with self- or relative-reported memory problems could be prescribed an app (neotivCare app [neotiv GmbH]) for comprehensive cognitive testing. Patients used the app to test their episodic memory function weekly for 12 weeks at home. After the testing period and the final consultation, physicians and patients received questionnaires to assess the app’s acceptance.

**Results:**

We received completed questionnaires from physicians for 45 patients. In addition, we received 45 completed questionnaires from the patients themselves. The physicians reported that, for most patients, the app supported their decision-making in the diagnostic process (26/45, 58%). In addition, most physicians found the app’s information dependable (34/45, 76%) and felt more certain in their decisions (38/45, 84%). From the patients’ perspective, a majority felt thoroughly tested (34/45, 76%), and only a few considered the time commitment for the cognitive tests to be too burdensome (7/45, 16%). Furthermore, despite the weekly cognitive testing and the lengthy 12-week testing period, a majority of patients participated in all tests (39/54, 72%).

**Conclusions:**

Our results indicate a high level of acceptance by physicians and patients, suggesting significant potential for the implementation of unsupervised digital cognitive assessments into routine health care. In the future, acceptance should be assessed in large-scale studies, with a particular focus on the impact on health care delivery and patient outcomes.

## Introduction

In many countries, the number of people with Alzheimer disease and related dementias is rapidly increasing; today, more than 50 million people are living with dementia, and this number is expected to triple by 2050 [1,2]. Timely diagnosis of Alzheimer disease is a key objective, as interventions and novel pharmacological treatment approaches shift to the disease’s early stages [3,4]. It is the primary care physicians who often have the closest contact with their patients and who serve as the patient’s initial contact point when they seek clarification for perceived changes in cognitive abilities. However, conventional neuropsychological screening tests lack sensitivity for early stages of Alzheimer disease [5]. The drawback of more comprehensive cognitive test batteries is that these tests are time-consuming and that they need to be administered by specialized personnel, thus they are quite resource-intensive [6]. This makes it challenging to integrate early diagnosis into primary care and outpatient practices, impacting access, especially in rural areas where the nearest memory clinic is often more distant. Another disadvantage is the 1-time testing in the setting of a medical practice, which has been shown to be influenced by daily variations (eg, lack of sleep) [7] and even the stereotype threat effect [8], reducing reliability and validity of the assessment.

A promising solution lies in digital cognitive assessments at home, which can save time and resources. Furthermore, they offer the possibility for repeated testing, reducing the impact of daily variations and allowing for the assessment of symptom progression over time. In recent years, several mobile apps for cognitive testing for early Alzheimer symptoms have been developed [9,10]. Among them is the neotivCare app (neotiv GmbH), which has been developed based on current insights into the functional anatomy of episodic memory [11-13]. The cognitive tests have already been assessed for their psychometric quality and feasibility in cognitively healthy older adults and in a memory clinic sample [6,14,15]. For other unsupervised mobile apps, feasibility and acceptance have been explored as well [e.g. 16,17].

What is currently lacking and of particular importance is testing the acceptance in the actual health care setting, for which this and other apps have been developed. In this study, participating physicians in outpatient care had the opportunity to prescribe the neotivCare app to patients who consulted them for memory problems. After the patients had used the app for a duration of 3 months, and once the physicians had received and analyzed the test results, the physicians then evaluated the usefulness of the app in the diagnostic process, and the patients provided their feedback on their experience with the app-based testing.

The objective of this study was to evaluate the acceptance of unsupervised app-based cognitive testing within the realm of care from both the physicians’ and the patients’ perspective. This study adheres to the Guidelines and Checklist for the Reporting on Digital Health Implementations (iCHECK-DH) [18].

## Methods

### Study Procedure and Questionnaires

The project took place in collaboration between the physicians’ network in a neighbourhood in the north-east of Germany (Magdeburg-Schönebeck), a major statutory health insurance in Germany (AOK Saxony-Anhalt), and neotiv GmbH. The study was conducted to test improved diagnostic methods for dementia care according to §140a “Special Care” of the German Social Code Book V. The study was conducted between September 2021 and December 2022 in Magdeburg-Schönebeck. This period coincided with social distancing measures due to the SARS-CoV-2 pandemic.

Participating practices were recruited from the members of the physicians’ network Magdeburg-Schönebeck. Patients consulting their physician for memory problems and who fulfilled further inclusion criteria were informed about the opportunity to test their cognition with the neotivCare app for 3 months. Inclusion criteria were self- or relative-reported memory problems persisting for at least 6 months and perceived as progressive, as well as owning a smartphone and being able to use it. In addition, patients had to be insured with a specific major statutory health insurance in Germany (AOK Saxony-Anhalt). The exclusion criterion was a clear indication of dementia, as it requires timely optimal care. After providing informed consent, patients received an activation code to install and use the app. The testing period spanned 12 weeks with weekly assessments. An automated report was generated from the test results and made available to the treating physician.

After the physician and patient had discussed the results, both the physician and the patient received a short questionnaire, the items are displayed in Table 1. The physician answered, for each patient individually, (1) to what extent the test results from the app had supported decision-making in the diagnostic process, (2) how they assessed the dependability of the app information for deciding on the further diagnostic process, and (3) how certain they were, based on the app information, in having made the correct decision about the subsequent process.

Patients completed a questionnaire providing sociodemographic information (age, sex, and educational attainment). In addition, they answered (1) to what extent they felt their cognitive abilities were thoroughly assessed through the app-based assessment and (2) to what extent they felt the assessments were too time-consuming for them.

**Table 1.** Description of the items answered by physicians and patients regarding app-based cognitive testing for early symptoms of Alzheimer disease in this implementation study in outpatient care.

Item		Response options
**Physician questionnaire**		
	How much did the app information provided about the patient support your decision regarding the further diagnostic process?	Strongly Rather strongly Rather limited Not at all
	How do you assess the dependability of the app information provided for deciding on the further diagnostic process?	Dependable Rather dependable Rather undependable Undependable
	How certain were you that, based on the app information provided about the patient, you made the right decision for the further process?	Certain Rather certain Rather uncertain Uncertain
**Patient questionnaire**		
	The regular memory assessments with the neotivCare app were, in terms of timing, for me:	Very burdensome Quite burdensome Minimally burdensome Not burdensome
	Through the regular memory assessments with the neotivCare app, I felt that I was thoroughly examined.	Fully applies Applies more Applies less Does not apply

### Ethical Considerations

Before the commencement of the study, the study was first ethically reviewed and approved by the ethical board of the physicians’ network Netz Magdeburg Schönebeck (Network Magdeburg Schönebeck), an association of resident physicians that participated in this study. Ethics approval was granted (no reference number) and a written statement confirming the approval is available. However, this statement is dated after the study's commencement, as the authors requested documentation of the prior ethical review. The patients were thoroughly informed about the study by their physician and through a patient information sheet. They provided informed consent through a participation declaration (Multimedia Appendix 1) before receiving an activation code to install and access the neotivCare app. The study reports results based on returned questionnaires regarding app evaluation; all questionnaires were anonymous. Linkage between the questionnaires, with medical records or with the results from the neotivCare app, was not possible. The participating physicians were compensated by the collaborating health insurance for their additional efforts, such as informing and enrolling patients in the study. The patients did not receive any compensation for their participation in the app-based cognitive testing. The results, figures, or supplements reported in the manuscript do not allow any identification of individual study participants.

### Sample

#### Medical practices

A total of 19 physicians from 15 medical practices participated in the study, including 2 (11%) specializing in internal medicine and 2 (11%) in neurology, with the remaining participating physicians being general practitioners (15/19, 79%). Physicians provided anonymized questionnaire responses on their acceptance of the app in the diagnostic process for 45 patients.

#### Patients

A total of 54 patients were included in the study. Of these, 45 individuals completed a questionnaire on sociodemographic factors and app usage. Table 2 presents the descriptive statistics for the study participants. The average age was 66 (SD 7.2, age range 52-80) years, 56% (25/45) were female. The majority had a moderate level of education (23/45, 51%).

**Table 2.** Description of the sociodemographic characteristics of the patients who participated in app-based cognitive testing for early symptoms of Alzheimer disease in this implementation study in outpatient care.

Variable		Value (N=45)
Age (years), mean (SD)		66.4 (7.2)
**Sex, n (%)**		
	Female	25 (56)
**Education, n (%)**		
	Low (<10 years of schooling)	11 (24)
	Medium (10‐11 years of schooling)	23 (51)
	High (≥12 years of schooling)	9 (20)
	Did not disclose	2 (4)

### The neotivCare App

Cognitive testing using the neotivCare app was conducted remotely, without supervision, and in a home setting. The patients used their own mobile phone or tablet. The tests were administered weekly over a period of 12 weeks. To remind patients to participate in the weekly test, they received a push notification when a test was available. If a patient did not start the test, they were reminded up to 5 more times via push notification (once a day for 5 days). To boost motivation for regular participation, study teams were trained to inform patients at enrollment that correct test execution and adherence to the study schedule are essential for generating valid data about their cognitive status.

**Figure 1.**
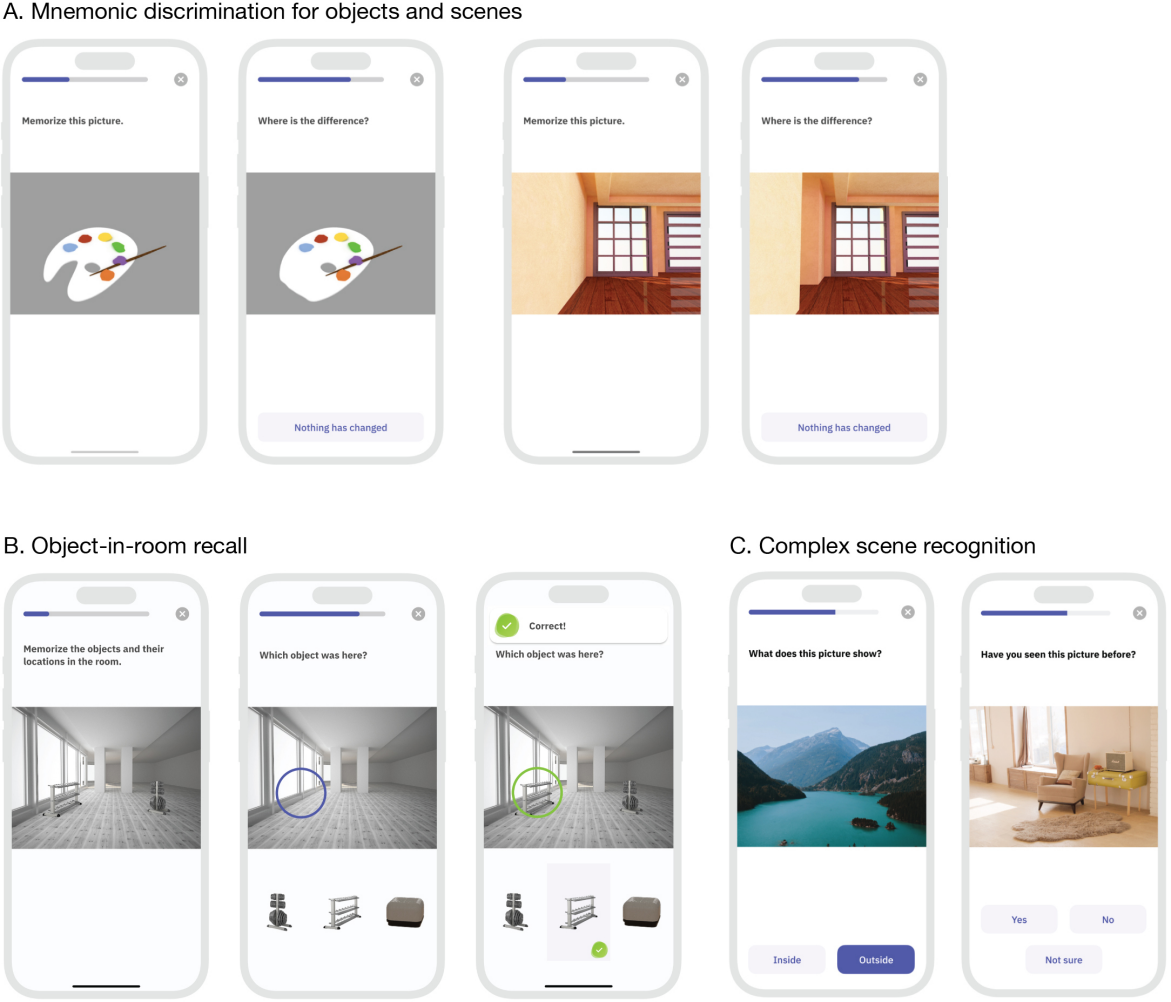
Illustration of the tasks used in app-based cognitive testing for early symptoms of Alzheimer disease in this implementation study in outpatient care (used with permission from neotiv GmbH).

A total of 3 different tasks were presented to assess episodic memory function. Each task was presented 4 times, resulting in the repetition of each specific task every 3 weeks. In the first task, patients were asked to indicate whether an image was an identical repetition or a modified version of a previous image and tap on the location of change (Mnemonic discrimination for objects and scenes, Figure 1A). In the second task, participants had to memorize objects and their positions within a room. Subsequently, they were shown an empty room with a blue circle highlighting the previous position of one of the objects and they had to select the correct object from a set of options (Object-in-room recall, Figure 1B). In the third task, patients were shown photographic images and asked to determine whether they depicted indoor or outdoor scenes. After 60‐70 minutes, images were presented again, and patients had to decide whether they were new or familiar (Complex scene recognition, Figure 1C).

### Statistical Analysis

The processing and analysis of the data were outsourced as an external contract and conducted by an independent research group. The analysis of responses to the items was done descriptively (relative frequencies).

### Comparison With Previous Work

A literature search was conducted to identify studies on the feasibility and acceptance of unsupervised digital cognitive assessments in health care and to compare them with our findings.

## Results

### Acceptance Among Outpatient Physicians

**Figure 2.**
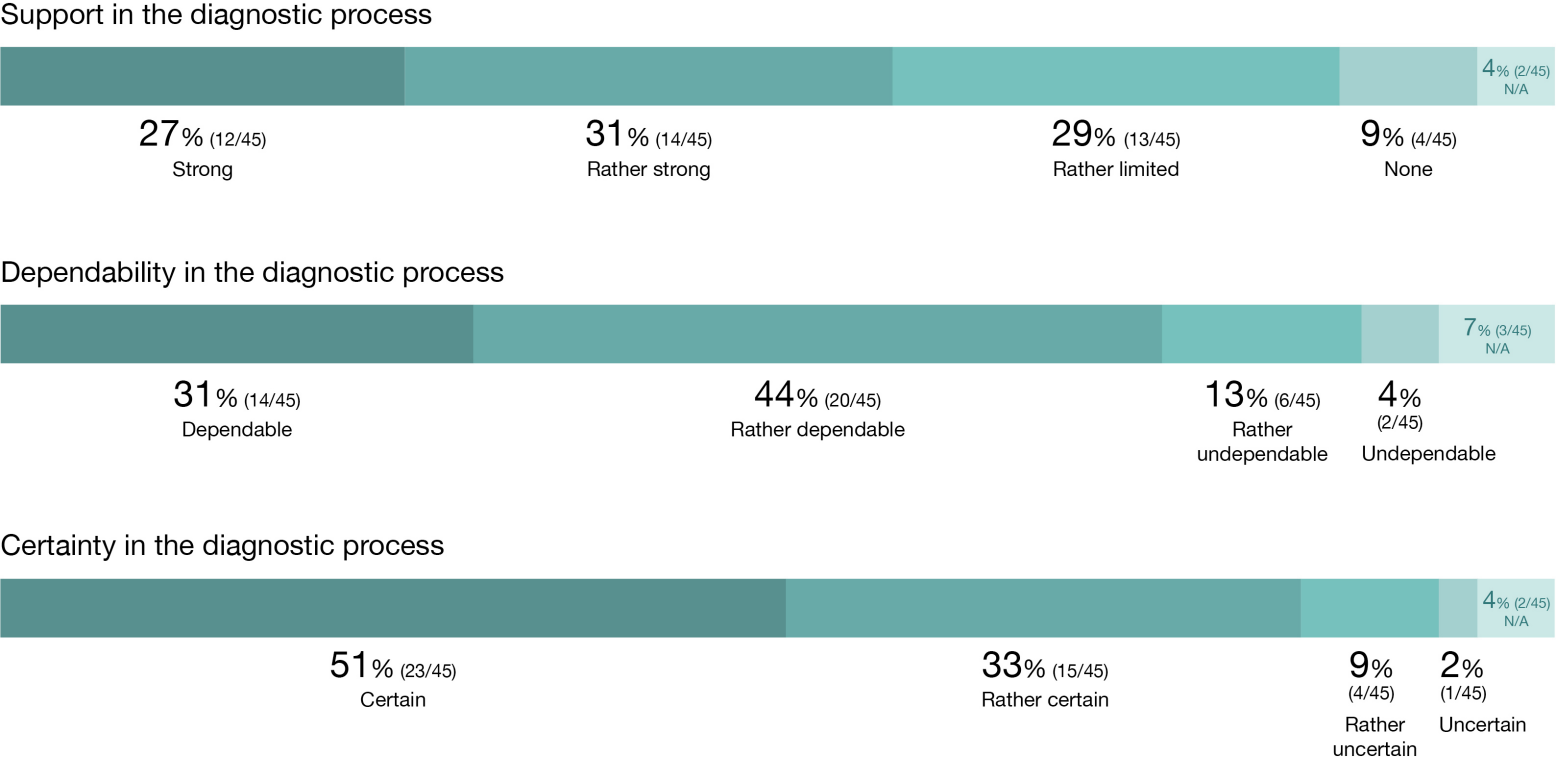
Distribution of responses from the physicians concerning acceptance of app-based cognitive testing for early symptoms of Alzheimer disease in this implementation study in outpatient care (data based on questionnaires from the 15 participating practices regarding 45 patients). N/A: not available.

Figure 2 shows the distribution of responses from the participating physicians. For the majority of patients, the physicians indicated that the information from the app supported their decision-making in the diagnostic process (strongly: 12/45, 27%; rather strongly: 14/45, 31%), with only a few cases where the app was perceived by physicians as not supporting the diagnostic process at all (4/45, 9%).

For a clear majority of patients, the physicians assessed the app information as dependable for making decisions about the further diagnostic process (dependable: 14/45, 31%; rather dependable: 20/45, 44%), with only a few patients where the app was considered somewhat (6/45, 13%) or not dependable at all (2/45, 4%).

In the clear majority of cases, the treating physicians reported feeling certain (23/45, 51%) or rather certain (15/45, 33%) in having made the correct decision for the further diagnostic process based on the app information. Only in a small number of cases did the physicians indicate that they were somewhat uncertain (4/45, 9%) or uncertain (1/45, 2%).

### Acceptance Among Patients

**Figure 3.**
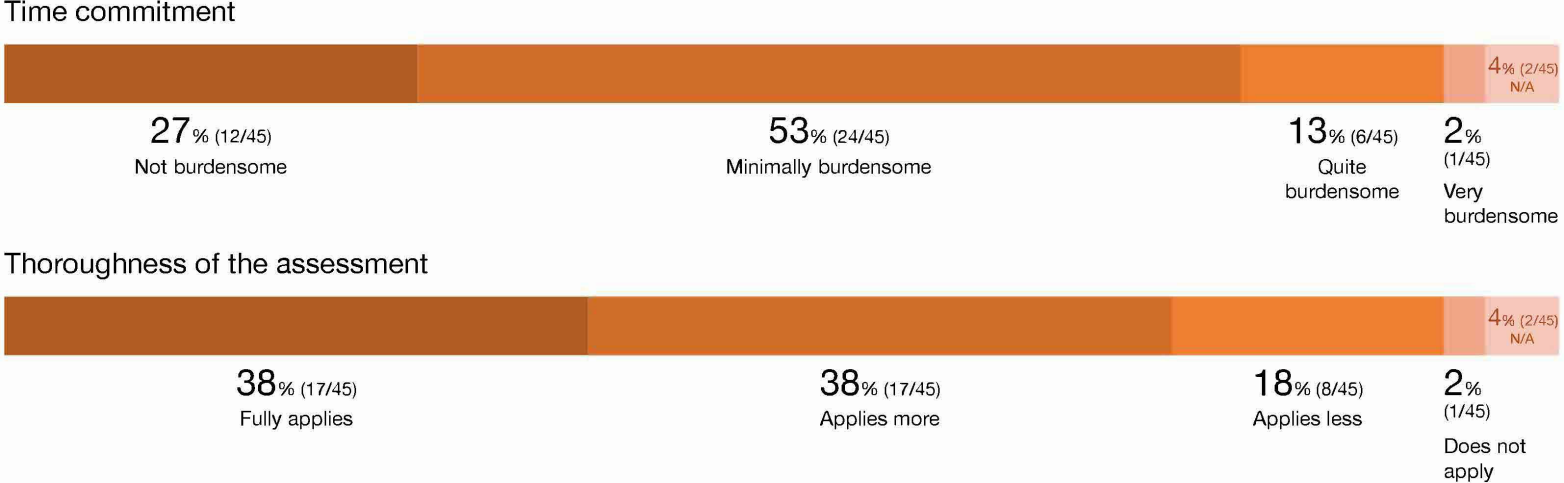
Distribution of responses from the patients concerning acceptance of app-based cognitive testing for early symptoms of Alzheimer disease in this implementation study in outpatient care (data based on responses from 45 of the 54 included patients). N/A: not available.

Figure 3 shows the distribution of responses from the participating patients. Of the patients who had started participating in the study, 72% (39/54) completed all weekly tests over 12 weeks, indicating high adherence throughout the study period.

When asked about the extent to which using the app was time-consuming, a clear majority responded that they felt either not at all (12/45, 27%) or only slightly (24/45, 53%) time-burdened by using the app. Feeling quite or very burdened was reported by 13% (6/45) and 2% (1/45) of the patients.

A large majority of patients stated that they felt thoroughly examined through the app; 38% (17/45) agreed and another 38% (17/45) somewhat agreed. Slightly more than one-fifth disagreed to some extent (8/45, 18%) or completely (1/45, 2%).

## Discussion

### Principal Results

In this implementation study of unsupervised app-based cognitive assessment in outpatient care, both physicians and patients showed high levels of acceptance. The physicians reported, for the majority of patients, that the app had supported the diagnostic process. The majority of patients found the repeated assessments to be only minimally burdensome and reported feeling thoroughly assessed. Most patients participated in all the weekly assessments over the 12-week testing period.

### Comparison With Previous Work

Previous studies have reported high feasibility for unsupervised digital cognitive assessments [9]. Several studies have documented high consent rates during recruitment; however, these were from ongoing studies, with the majority of participants approached agreeing to additional digital cognitive testing [19-21]. Adherence rates in previous studies were also high, with most participants completing multiple assessments, ranging from several days [22] to several months [23]). Unsupervised digital cognitive assessments have been tested both in the general population [14] and among older adults [6].

What is currently lacking and urgently needed are studies that test unsupervised digital cognitive assessments within the realm of health care [24,25]. Particularly important in this context is the question of acceptance by physicians and patients, as physicians are responsible for prescribing, interpreting, and acting on these assessments, while patients are required to thoroughly complete the cognitive tests. To our knowledge, there are no published results on this topic and this study is the first to report on the acceptance of such assessments by physicians and patients.

### Physician and Patient Perspectives Regarding App-Based Cognitive Assessment

Physicians in our study indicated that, for most patients, the app supported them in making decisions about the diagnostic process. Furthermore, they indicated that they perceived the app information as dependable and that they felt more certain in their decisions. These initial findings suggest that participating physicians have shown acceptance toward the app for evaluating cognitive performance and were using the results to inform their next steps. This is promising, as acceptance from outpatient care physicians is crucial for integrating such tools into routine care and enhancing early testing for Alzheimer disease, as they often serve as the first point of contact for individuals experiencing memory problems.

The participating patients also gave largely positive feedback on the app; a majority felt thoroughly tested, and only a few considered the time commitment for the cognitive tests as too burdensome. Furthermore, despite weekly cognitive testing and the lengthy testing period of 12 weeks, a large majority of patients participated in all tests, indicating high adherence. Reasons for this likely included the generally high motivation of patients in the context of assessing a potential Alzheimer disease diagnosis. In addition, the study design and app programming were aimed at enhancing adherence—study teams were trained to inform patients at enrollment about the necessity of correctly performing tests and adhering to the study plan to generate valid data for the diagnostic report. Furthermore, the app sent weekly push notifications (and reminders) when a new test was available.

These initial results indicate a high level of acceptance and adherence from the patients. This is an important finding for the implementation of app-based cognitive assessment into routine health care, given that it is ultimately the patients who need to integrate the tests into their daily lives and perform them without supervision over an extended time period.

### Limitations

To ensure data protection, it was not possible to link the physicians’ and patients’ questionnaires with each other, nor was it possible to link them with results from the app or additional patient data. The results presented here are solely derived from the returned questionnaires, which were anonymized and provided to participating physicians and patients. The overall sample size was relatively small, and the inclusion criteria of the study may have introduced a potential selection bias in the recruitment process. For example, only patients who owned and could use a smartphone or tablet were included. However, currently, a majority of people aged 55-65 years already own a smartphone, and this number is expected to rise in the future [26]. Furthermore, only patients insured with a specific major public health insurance (AOK Saxony-Anhalt) were eligible to participate. However, as the largest statutory health insurance in Saxony-Anhalt, it covers a cross-section of the population.

### Conclusion

The high acceptance and adherence observed in our study is a promising finding for the implementation of unsupervised digital cognitive assessments within the realm of care. Integrating these assessments into routine health care has the potential to enhance early detection of Alzheimer disease, improve access to diagnostics in rural areas, and reduce the burden on medical practices. Future research should focus on larger-scale studies to confirm the high acceptance rates and evaluate the benefits of unsupervised digital cognitive assessments in health care delivery and their impact on patient outcomes.

## Supplementary material

10.2196/62706 Multimedia Appendix 1 Participant declaration.

## Abbreviations

iCHECK-DH: Guidelines and Checklist for the Reporting on Digital Health Implementations
