# Supplementary material for: Acceptance of Unsupervised App-Based Cognitive Assessment in Outpatient Care: An Implementation Study
Source: JMIR Form Res. 2025 Feb 13;9:e62706. doi: 10.2196/62706 (PMC11841747; doi:10.2196/62706)
Supplement: Multimedia Appendix 1 [file formative-v9-e62706-s001.pdf]

Krankenkasse bzw. Kostenträger		
Name, Vorname des Versicherten<br>geb. am		
Kostenträgerkennung	Versicherten-Nr.	Status
Betriebsstätten-Nr.	Arzt-Nr.	Datum

Teilnahmeerklärung und Einwilligung zur Datenverarbeitung Anlage 1

Vertrag über die Erprobung der App „neotivCare“ zur verbesserten Diagnostik Patienten mit selbstberichteten oder vermuteten Gedächtnisproblemen gem. § 140a SGB V 12088111178

Verteiler:

□ Info an die AOK Sachsen-Anhalt/ Fax 0391 2878 44570
□ Bestandteil der Patientendokumentation des Arztes
□ Ausfertigung Patient

- **Teilnahmeerklärung**

Hiermit erkläre ich, dass

- ich bei der AOK Sachsen-Anhalt versichert bin, mein Wohnort in Sachsen-Anhalt liegt und über 18 Jahre alt bin.
- ich die Versicherteninformation zur Teilnahme an der besonderen Versorgung erhalten, gelesen und zur Kenntnis genommen habe.
- ich umfassend über Wesen, Bedeutung und Tragweite der besonderen Versorgung nach § 140a SGB V und die Bedingungen zur Teilnahme aufgeklärt wurde. Alle Fragen wurden zu meiner Zufriedenheit beantwortet.
- der unterzeichnende Arzt mein gewählter Arzt ist.

Mir ist bekannt, dass

- die Teilnahme am Vertrag freiwillig ist und **innerhalb von 2 Wochen ohne Angabe** von Gründen schriftlich, elektronisch oder zur Niederschrift gegenüber der AOK Sachsen-Anhalt **widerrufen** werden kann. Zur Fristwahrung genügt das rechtzeitige Absenden der Erklärung an die AOK Sachsen-Anhalt.
- mein Arzt des Arztnetzes bei meiner besonderen Versorgung zusätzliche Leistungen erbringt. Dies umfasst die Durchführung der Diagnostik, inklusive der Anleitung zur Nutzung der App, die Entgegennahme der Ergebnisse und das Auswertungsgespräch.
- ich am Ende der Nutzungszeit dem Arzt die Ergebnisse für das Auswertungsgespräch übermitteln muss und ich gebeten werde, einen Fragebogen zur App auszufüllen.

Datenschutzrechtliche Einwilligungserklärung

Die von meiner Krankenkasse und den Vertragspartnern in der Versicherteninformation (diesem Formular beigefügt) beschriebenen Regelungen zur Erhebung, Verarbeitung und Nutzung meiner Daten (im Weiteren nur Datenverarbeitung) auf Grundlage des Vertrages, habe ich zur Kenntnis genommen und bin mit der beschriebenen Datenverarbeitung im Rahmen meiner Teilnahme einverstanden. Weiter ist mit bekannt, dass die beteiligten Ärzte, die jeweils von Ihnen erhobenen Daten zur optimalen Abstimmung meiner Behandlung im erforderlichen Umfang verarbeiten (z.B. übermitteln) und nutzen und dabei die geltenden gesetzlichen Bestimmungen zum Umgang mit ärztlicher Schweigepflicht, dem Sozialgeheimnis und den datenschutzrechtlichen Vorschriften gewahrt bleiben. Sofern ich keine Einwilligung zur Datenverarbeitung für diesen besonderen Versorgungsvertrag erteile, ist eine Teilnahme an diesem besonderen Angebot nicht möglich. In diesem Fall ändert sich aber an der bisherigen ärztlichen Versorgung für mich nichts.

Widerspruchsrecht

Bei der Datenverarbeitung auf Grund einer Einwilligung besteht das Recht, diese jederzeit mit **Wirkung für die Zukunft** zu widerrufen. Der Widerruf berührt nicht die bis zum Zeitpunkt des Widerrufs verarbeiteten Daten.

Eine zukünftige Teilnahme an dem Vertrag ist jedoch nicht mehr möglich.

☐ Evaluation: **Ja**, ich bin mit der Erfassung und wissenschaftlichen Auswertung, wie in der Versicherteninformation zur Datenverarbeitung beschrieben, einverstanden.

Insbesondere ist mir bekannt, dass bei der Durchführung und Dokumentation der Behandlung sowie bei der Weitergabe von Verwaltungsdaten und medizinischen Daten die ärztliche Schweigepflicht, das Sozialgeheimnis und die datenschutzrechtlichen Vorschriften gewahrt bleiben.

- nur vom Versicherten (bzw. gesetzliche Vertreter) auszufüllen **-**

Ja, ich möchte an dieser besonderen Versorgungsform unter Verarbeitung meiner Daten im erforderlichen Umfang durch die Vertragspartner **teilnehmen** und bestätige dies mit meiner Unterschrift.

Datum

Unterschrift des Versicherten bzw. gesetzlichen Vertreters

- nur vom Arzt auszufüllen **-**

Datum

Ich bestätige, dass der o. g. Patient die Einschreibevoraussetzungen zur Teilnahme an diesem Vertrag erfüllt.

Unterschrift/Vertragsarztstempel
